# Nitrogen and sulfur metabolisms encoded in prokaryotic communities associated with sea ice algae

**DOI:** 10.1038/s43705-023-00337-2

**Published:** 2023-12-11

**Authors:** Christopher M. Bellas, Karley Campbell, Martyn Tranter, Patricia Sánchez-Baracaldo

**Affiliations:** 1 Department of Ecology, Universität Innsbruck, 6020 Innsbruck, Austria; 2 Department of Arctic and Marine Biology, UiT The Arctic University of Norway, Tromsø, Norway; 3 Bristol Glaciology Centre, School of Geographical Sciences, University of Bristol, Bristol, UK; 4 Arctic Research Centre, Department of Bioscience, University of Aarhus, Aarhus, Denmark

**Keywords:** Water microbiology, Metagenomics

## Abstract

Sea ice habitats harbour seasonally abundant microalgal communities, which can be highly productive in the spring when the availability of light increases. An active, bloom-associated prokaryotic community relies on these microalgae for their organic carbon requirements, however an analysis of the encoded metabolic pathways within them is lacking. Hence, our understanding of biogeochemical cycling within sea ice remains incomplete. Here, we generated metagenomic assembled genomes from the bottom of first-year sea ice in northwestern Hudson Bay, during a spring diatom bloom. We show that the prokaryotic community had the metabolic potential to degrade algal derived dimethylsulphoniopropionate and oxidise sulfur. Facultative anaerobic metabolisms, specifically, dissimilatory nitrate reduction and denitrification were also prevalent here, suggesting some sea ice prokaryotes are metabolically capable of adapting to fluctuating oxygen levels during algal bloom conditions. Such denitrification could be an important loss of fixed-N_2_ in the changing Arctic marine system.

## Introduction

Sea ice creates a dynamic habitat for microorganisms, controlled by fluctuations in light, salinity and temperature caused by seasonal extremes, combined with localised variability in atmosphere-ice-ocean gas and nutrient exchange. An area of particularly high seasonal productivity is the bottom centimetres of the ice, where an abundant microalgal community develops in spring, dominated by diatoms [1]. This microalgal growth accelerates when light conditions are optimal and is supported by nutrients predominantly coming from the water column [2]. Diatom blooms release extracellular polymeric substances (EPS) into their surroundings to increase sea ice habitability [3] and the sulfur metabolite dimethylsulfoniopropionate (DMSP) [4, 5], which acts as an osmoregulator and cryoprotectant [6]. There is evidence that DMSP production varies with the composition of cold-adapted algal blooms, with species like *Nitzschia* spp. having driven its release in previous studies of sea ice [4]. Both EPS and DMSP can be readily metabolised by the local prokaryotic community [3, 4, 7], with DMSP representing a major source of reduced sulfur to marine prokaryotes [8, 9], although the individual species involved and metabolic pathways remain unclear for sea ice habitats. This active heterotrophic community has the potential to create localised oxygen limited environments [10, 11]. Indeed, sulphate reducing bacteria, such as *Desulforhopalus* (Deltaproteobacteria), have been found in Antarctic sea ice [12], and anaerobic denitrification processes have been detected in Arctic sea ice [11, 13].

In this study, we analyzed the prokaryotic communities associated with a diatom bloom in sea ice (*n* = 4) from northwestern Hudson Bay, to reveal a proportion of the prokaryotic community encoded for facultatively anaerobic processes. We also identified DMSP degradation and sulfur oxidation genes in dominant taxa, suggesting that algal derived DMSP is an important energy source for sea ice prokaryotes.

## Results and discussion

### Sea ice algal composition

**Fig. 1:**
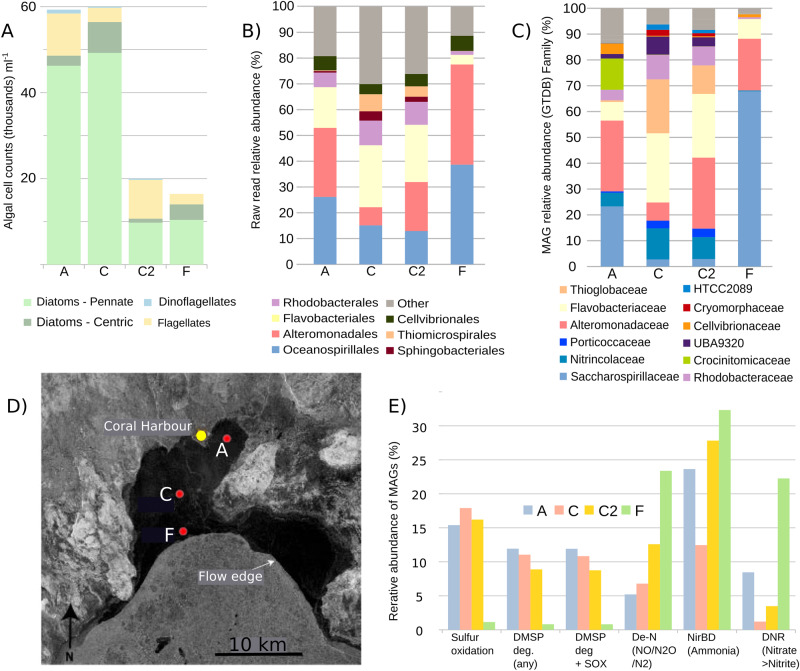
Microbial diversity across a sea ice transect in northwestern Hudson Bay, May 2019. Metagenomic Assembled Genomes (MAGs) were generated from the bottom 10 cm of the sea ice. **A** Algal cell counts (thousands ml^−1^) in melted ice. **B** Relative abundance of prokaryotic orders based on 16S rRNA reads extracted from unassembled metagenomic data (Silva database). **C** Relative abundance and taxonomy (Genome Taxonomy Database - GTDB) of top 104 MAGs which were used in the metabolic pathway analysis. The 104 MAGs together recruited 25–50% of all metagenomic reads and, based on 16S rRNA reads, were a broadly representative subset of prokaryotic taxa from which to investigate the metabolic functions. **D** Map of sample sites in relation to Coral Harbour and the flow edge, site C was sampled on two occasions 17/5/2019 (C1) and 30/05/2019 (C2). **E** Predicted metabolic pathways encoded in MAGs by comparison with the KEGG database. The y-axis shows the relative abundance of all MAGs encoding each specific metabolic pathway (reads mapped as a percentage of all MAG mapping reads): Sulfur oxidation - any complete sulfur oxidation pathway (see Fig. 2 for list); DMSP deg. - DMSP degradation genes including DMSP lyases and/or DMSP demethlyase; DMSP deg. + SOX - MAGs with a combination of DMSP degradation pathways or complete sulfur oxidation pathways as above; De-N - MAGs encoding a denitrification pathway including nitrite reductases (nirS/nirK), nitric oxide reductase (norBC), nitrous oxide reductase (nosZ); NirDB - nitrite reductase to ammonia; DNR - dissimilatory nitrate reduction (nitrate reductases; napAB or narB).

Significant algal growth was documented in the bottom-ice at all sites along a transect to the ice flow edge (16,000–60,000 cells ml^−1^). The community was dominated by pennate diatoms (Fig. 1A, D), specifically *Nitzschia* spp. (Site A and C) and *Entomoneis kjellmanii* (Site F), which had a relative abundance of 63–82% of the community [4].

### Prokaryotic diversity

**Fig. 2:**
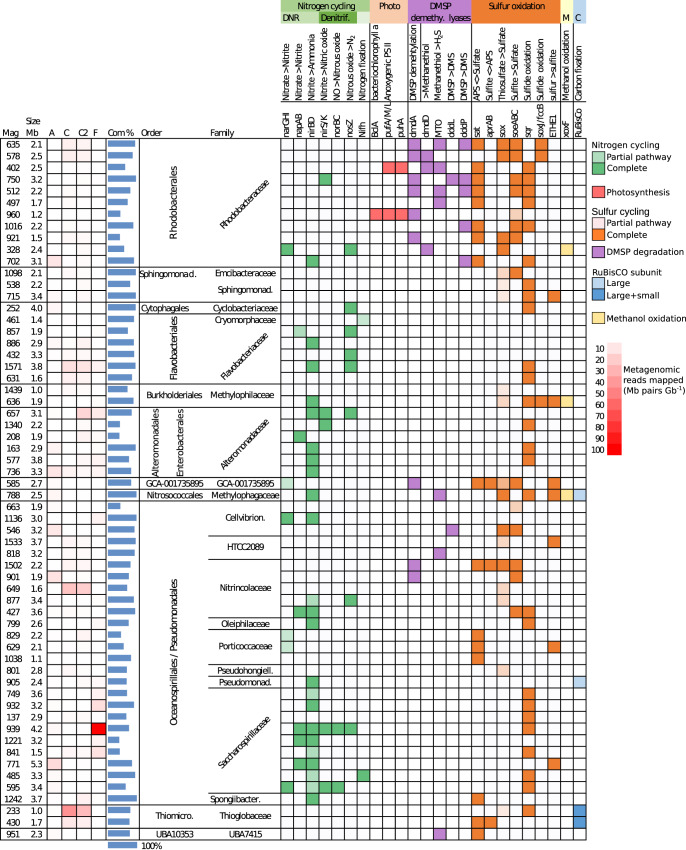
Selected MAGs from the dataset which were functionally annotated with genes involved in sulfur and nitrogen cycling. MAG - MAG number; Mb - MAGs size in Mb pairs; Relative abundance heatmap - Mb pairs per Gb (see key on right); Com - genome completeness estimate; DNR - Dissimilatory Nitrate Reduction, consisting of narGHI - nitrate reductase alpha, beta and gamma subunits; napAB - nitrate reductase subunits; nirBD - nitrite reductase subunits; Denitrification pathway consisting of nirS/nirK - nitrite reductases (NO - forming); norB/norC - nitric oxide reductase subunits; nosZ - nitrous oxide reductase. Nitrogen fixation - consisting of nifH (nitrogenase). Photo - bacterial photosynthetic genes consisting of bclA - bacteriochlorophyll a; pufA/pufM/pulL - light-harvesting complex 1 alpha chain/photosynthetic reaction center M and S subunits. DMSP demethy. - dimethylsulfoniopropionate demethylation pathway genes consisting of dmdA (DMSP demethylase) and dmdD ((methylthio)acryloyl-CoA hydratase) which forms methanethiol; MTO - methanethiol oxidase. DMSP lyases - consisting of dddL/dddP/dddQ which form dimethyl sulfide (DMS). Sulfur oxidation - genes involved in sulfur oxidation via reverse-acting dissimilatory sulfate reduction, encoded by sat (sulfate adenylyltransferase) and aprAB (adenylylsulfate reductase subunits); sulfate oxidation via the Sox multi enzyme system consisting of the complete group SoxXYZABCD; soeABC - sulfite dehydrogenase subunits; sqr - sulfide:quinone oxidoreductase; soxJ/fccB - sulfide dehydrogenase; ETHE1 - sulfur dioxygenases. Methanol oxidation - xoxF - methanol dehydrogenase.

Associated with the bloom was a diverse prokaryotic community, dominated by the orders Oceanospirillales (NCBI) / Pseudomonadales (GTDB), Alteromonadales (NCBI) / Enterobacterales (GTDB), Flavobacteriales and Rhodobacterales (Fig. 1B, C), which together accounted for 56–83% of prokaryotic diversity by analysis of unassembled reads (Supplementary Methods). Previous 16S rRNA studies have detected dominant Alteromonadaceae and Flavobacteriaceae in sea ice [14, 15]. In terms of metabolic functions, 14 out of 104 MAGs (1–12% of the MAGs by relative abundance) (Fig. 1E; Supplementary Table 1) encoded genes for DMSP degradation, either through lysis to dimethylsulfide (DMS) (dddL or dddP genes), or though a demethylation pathway (dmdA or dmdD) to methanethiol [7, 16], with seven MAGs further encoding the ability to oxidise methanethiol to hydrogen sulfide. Nearly all DMSP degraders (13 of 14 MAGs), and an additional 10 MAGs (1–18% of all MAGs by relative abundance) encoded sulfur oxidation genes (Fig. 2), suggesting that sulfur from DMSP degradation is utilised for energy, either through DMSP demethylation and methanethiol oxidation, or another undetermined pathway. These genes were particularly prevalent in members of the Rhodobacteraceae family, some of which are well known to metabolise DMSP [17]. In previous research on Arctic frost flowers, DMSP catabolism genes were attributed to members of the *Rhizobiales* [18].

Facultatively anaerobic Dissimilatory Nitrate Reduction (DNR) to ammonia and denitrification genes were prevalent in many abundant members of the prokaryotic community. Eight MAGs (Fig. 2) encoded the ability to anaerobically respire inorganic nitrate (napAB or narGHI genes), these represented 1–22% of all MAGs by relative abundance (Fig. 1E). Six of these also encoded nitrite reductase (nirBD) genes, suggesting nitrite may be further reduced to ammonia. Two Saccharospirillaceae MAGs (MAGs 595 and 939; Fig. 2), representing up to 20% of MAGs by relative abundance in site F, additionally encoded denitrification pathways from nitrate to nitrous oxide or dinitrogen, with another nine MAGs (Alteromonadaceae, Nitrincolaceae, Saccharospirillaceae, Flavobacteriaceae and Rhodobacteraceae) encoding one or more genes from the denitrification pathway (nirS/K, norBC or nosZ), showing the potential for denitrification in 5–23% of the community by MAG relative abundance (Fig. 2). The ability to anaerobically respire oxidised forms of nitrogen may be particularly advantageous to sea ice prokaryotes, which are subjected to low or fluctuating oxygen conditions [10, 11].

The respiratory reduction for nitrate may also be coupled to the oxidation of reduced sulfur to support chemolithotrophic growth, evidence for this was found in three Rhodobacteraceae MAGs (Fig. 2), one of which (MAG 328) encoded three nitrate reductase subunits (narGHI) along with a complete set of sox genes (soxXYZABCD). Concurrently, five members of the Nitrincolaceae family were represented (0–12% by relative abundance), which are known to be dominant gammaproteobacteria associated with the termination phase of diatom blooms [19] and often involved in denitrification [20]. One of these MAGs encoded a complete pathway for the dissimilatory reduction of nitrate to ammonia (napAB and nirBD) along with the soeABC enzyme complex, but without detectable CO_2_ fixation genes (RuBisCO), suggesting a chemoheterotrophic lifestyle. All Nitrincolaceae MAGs contained complete or partial pathways for sulfur oxidation, either via the soeABC enzyme complex, reverse-acting dissimilatory sulfate reduction (sat/aprAB) or the sox system, indicating that these organisms were all involved in sulfur cycling. Interestingly, members of the Thiomicrospirales were well represented by two partial MAGs (*Thioglobus* spp., MAGs 233 and 430; 0.1–21% of MAG relative abundance). Thiomicrospirales are typical oxygen minimum zone taxa [21], with *Thioglobus* spp. belonging to the SUP05 clade of chemoautotrophs, known to couple sulfite oxidation to nitrite reduction [22]. From our KEGG analysis, one *Thioglobus* sp. contained reverse-acting dissimilatory sulfate reduction genes (aprAB), whilst the other encoded a Sulfide:Quinone Oxidoreductase. Both encoded RuBisCO, which suggests a chemolithoautotrophic lifestyle, however, no dissimilatory nitrate reduction genes were identified, which may have been because of the incomplete nature of these MAGs (73–75% estimated by CheckM).

## Conclusions

This study highlights a heterogeneous sea ice prokaryotic community which is metabolically capable of sea ice algal DMSP degradation and associated sulfur oxidation, whilst also encoding for facultatively anaerobic metabolisms. These findings, combined with previous observations for anoxic conditions and denitrification, show that sea ice prokaryotic communities have the potential to maintain metabolic activity under fluctuating oxygen levels and influence nutrient cycles. Whilst we show genomic potential, we cannot comment on the activity of such processes. Further investigations are now needed to characterise the activity of these metabolic pathways in concert with the extent and variability of oxygen concentrations in sea ice. An understanding of sea ice denitrification is critical for our understanding of microbial production and nutrient limitation during algal blooms in this rapidly changing habitat.

### Supplementary information

Supplementary Text

Supplementary Table 1

## Data Availability

Raw reads are available in GenBank under BioProject PRJNA1011243, MAGs are found under BioSamples SAMN37641281-SAMN37641385. All assembled data are available from JGI (https://gold.jgi.doe.gov/projects) under GOLD Project IDs: Gp0507596 Gp0507597, Gp0507598, Gp0507599.
