# Supplementary material for: Nitrogen and sulfur metabolisms encoded in prokaryotic communities associated with sea ice algae
Source: ISME Commun. 2023 Dec 11;3:131. doi: 10.1038/s43705-023-00337-2 (PMC10713554; doi:10.1038/s43705-023-00337-2)
Supplement: Supplementary file 1 — Supplementary Text [file 43705_2023_337_MOESM1_ESM.docx]

**Supplementary Text:**

**Nitrogen and sulfur metabolisms encoded in prokaryotic communities associated with sea-ice-algae**

Christopher M Bellas^1^, Karley Campbell^2^, Martyn Tranter^3,4^, Patricia Sánchez-Baracaldo^3^

**Material and Methods**

**Sea ice sampling from Northwestern Hudson Bay**

Sea ice samples were collected offshore from the community of Coral Harbour, Nunavut, in northwestern Hudson Bay, in May 2019 during the course of the spring microalgal bloom. The land-fast sea ice in this study was 1.55 ± 0.12 m thick with 9-23 cm of snow cover (Table S1). Ice cores were taken using a 9 cm core barrel (Kovacs) from three study sites along a transect (Table S1) that was orientated perpendicular to the edge of a polynya and the coastline (Figure 1). The sites (A, C, and F) were situated increasingly offshore from Coral Harbour. Following collection, the bottom 10 cm of ice cores were individually melted and pre-filtered through a 10 µm filter, before concentration on a 0.2 µm polycarbonate filter to collect prokaryotes. Prior to filtration all tubing and filters were cleaned with 90% ethanol.

**Table S1** Location of ice cores drilled and size of metagenomes generated.

| Site | Location | Date | Snow depth (cm) | Chlorophyll a (mg m^-2^) | Metagenome  size (Gb pairs) |
| --- | --- | --- | --- | --- | --- |
| A | 64°06'52.6"N 83°04'45.8"W | 17/5/19 | 15 | 4.5 ± 2.0 | 11.4 |
| C | 63°59'39.1"N 83°20'19.1"W | 17/5/19 | 9 | 50.8 ± 6.0 | 12.1 |
| C2 | 63°59'39.1"N 83°20'19.1"W | 30/5/19 | 23.5 | 2.5 ± 0.2 | 11.9 |
| F | 63°54'36.7"N 83°18'24.6"W | 17/5/19 | 19 | 11.7 ± 2.0 | 13.5 |

**DNA extraction and sequencing:** DNA was extracted from four 47mm 0.2 µm polycarbonate filters using a DNeasy PowerWater Kit (Qiagen). Four sequencing libraries were prepared using the TrueSeq Nano DNA LT library prep kit and sequenced on an Illumina NextSeq 500 using 2 X 150bp paired end reads, generating ~ 40Gb of metagenomic data (Bristol Genomics Facility; http://www.bristol.ac.uk/biology/genomics-facility/). Reads were trimmed using Trimmomatic v0.39 (1) (settings: ILLUMINACLIP:TruSeq3-PE.fa:2:30:10 LEADING:3 TRAILING:3 SLIDINGWINDOW:4:15 MINLEN:36).

**Microbial diversity, 16S rRNA gene reads:** Metagenomic reads corresponding to 16S rRNA genes were extracted from each metagenome using Parrallel-META3 (2). Fasta reads of 150bp, were classified at 97% similarity to the Silva 16S database. Because of the short length, we restricted prokaryotic classification to the order level from this analysis.

**Metagenomic assembled genomes:** The four metagenomes (Table S1), were concatenated before being assembled using MEGAHIT v1.2.9 (3) (Settings: --k-list 21,29,39,59,79,99,119,141 --min-contig-len 200). For metagenomic binning, only contigs >7000bp were carried forward and used to generate contig coverage profiles. We mapped reads from each of the four individual metagenomes back to the assembly using Bowtie2 v2.2.6 (4) (settings: --sensitive --no-unal) and converted the output to BAM format using Samtools (5) (samtools view; samtools sort). The jgi_summarize_bam_cointig_depth program from the MetaBat2 suite (6) was used to create a depth file by calculating the mean read coverage death for each contig in each metagenomes (Settings: --percentidentity 97). The depth profile and assembled contigs file were then used as input into MetaBat2, to generate Metagenomic Assembled Genomes (MAGs).

**MAG analysis:** MAGs were assessed for completeness and contamination by CheckM (7) and taxonomically assigned using GTDB-Tk (8) on the Kbase platform (9). MAGs were assigned a quality score based on the estimated completeness and contamination (CheckM). High quality: >90% and < 5% contamination; Medium quality >50 % <10% Contamination; Low quality <50% < 15% contamination. Two mixed MAGs with relatively high contamination were included in the final analysis, as these represented abundant members of the community, which were composed of two similar strains which had been binned as one. Each of these MAGs had two copies of specific genes used in our analysis which were involved in nitrogen and sulfur cycling, meaning both strains encoded a copy of each functional gene, therefore we discuss these two mixed MAGs as one: MAG 771 (Saccharospirillaceae) was composed of two closely related genomes which could not be separated (50% contamination by CheckM). This MAG contained two copies of each of the genes responsible for dissimilatory Nitrate reduction to Ammonia (napAB and nirBD); MAG 430 (Thioglobaceae family; 30% contamination) possessed two copies of each gene in the reverse dissimilatory sulfate reduction pathway (sat and aprAB). As both MAGs had two copies of the respective functional genes, we were confident both strains in the mixed bins encoded these functions.

MAGs were annotated against the KEGG database using GhostKoala (https://www.genome.jp/kegg/kaas/). Functional pathways for nitrogen and sulfur cycling were assessed using the KEGG mapper tool, Reconstruct Pathway (10). Additional BLASTP searches were carried out to detect the DMSP lyase (dddP, E^-100^ cutoff). We calculated the relative abundance of a particular MAG by interrogating the BAM files to count the number of reads mapping to each individual MAG and expressed this as a percentage of the reads mapping to all 104 MAGs (relative MAG abundance). Because of ongoing reclassification of prokaryotic orders, we display orders in Figure 1 how they appear in the Silva database, with GDTB order in brackets.

**Supplementary data 1**

Excel file

**References**

1. Bolger AM, Lohse M, Usadel B. Trimmomatic: a flexible trimmer for Illumina sequence data. Bioinformatics. 2014 Aug 1;30(15):2114–20.

2. Jing G, Sun Z, Wang H, Gong Y, Huang S, Ning K, et al. Parallel-META 3: Comprehensive taxonomical and functional analysis platform for efficient comparison of microbial communities. Sci Rep. 2017 Jan 12;7(1):40371.

3. Li D, Liu CM, Luo R, Sadakane K, Lam TW. MEGAHIT: an ultra-fast single-node solution for large and complex metagenomics assembly via succinct de Bruijn graph. Bioinforma Oxf Engl. 2015 May 15;31(10):1674–6.

4. Langmead B, Salzberg SL. Fast gapped-read alignment with Bowtie 2. Nat Methods. 2012 Apr 1;9(4):357–9.

5. Li H, Handsaker B, Wysoker A, Fennell T, Ruan J, Homer N, et al. The Sequence Alignment/Map format and SAMtools. Bioinforma Oxf Engl. 2009 Aug 15;25(16):2078–9.

6. Kang DD, Li F, Kirton E, Thomas A, Egan R, An H, et al. MetaBAT 2: an adaptive binning algorithm for robust and efficient genome reconstruction from metagenome assemblies. PeerJ [Internet]. 2019 Jul 26 [cited 2021 Apr 28];7. Available from: https://www.ncbi.nlm.nih.gov/pmc/articles/PMC6662567/

7. Parks DH, Imelfort M, Skennerton CT, Hugenholtz P, Tyson GW. CheckM: assessing the quality of microbial genomes recovered from isolates, single cells, and metagenomes. Genome Res. 2015 Jul 1;25(7):1043–55.

8. Chaumeil PA, Mussig AJ, Hugenholtz P, Parks DH. GTDB-Tk: a toolkit to classify genomes with the Genome Taxonomy Database. Bioinformatics. 2020 Mar 1;36(6):1925–7.

9. Arkin AP, Cottingham RW, Henry CS, Harris NL, Stevens RL, Maslov S, et al. KBase: The United States Department of Energy Systems Biology Knowledgebase. Nat Biotechnol. 2018 Aug 1;36(7):566–9.

10. Kanehisa M, Sato Y. KEGG Mapper for inferring cellular functions from protein sequences. Protein Sci Publ Protein Soc. 2020;29(1):28–35.
